# Development and application of a rapid HPLC method for simultaneous determination of hyperoside, isoquercitrin and eleutheroside E in *Apocynum venetum* L. and *Eleutherococcus senticosus*

**DOI:** 10.1186/s13065-020-00687-1

**Published:** 2020-05-02

**Authors:** Jie Shen, Kai Yang, Cheng Jiang, Xiao-qiong Ma, Min-xia Zheng, Cai-hua Sun

**Affiliations:** 1 The First Affiliated Hospital of Zhejiang Chinese Medical University, No. 54 Youdian Road, Hangzhou, 310 006 Zhejiang Province China; 2 The First Affiliated Hospital of Medical School of Zhejiang University, Hangzhou, 310 003 Zhejiang Province China; 3 Department of pharmacy, Tongde Hospital of Zhejiang Province, Hangzhou, 310 012 Zhejiang Province China

**Keywords:** Hyperoside, Isoquercitrin, Eleutheroside E, Quality control

## Abstract

*Apocynum venetum* L. and *Eleutherococcus senticosus* have been used for hundreds of years to treat hypertension in China. In previous research, there was not a suitable quality control of method for the formulas of *Apocynum venetum* L. and *Eleutherococcus senticosus.* It is urgent and essential to develop modern analytical methods for *Apocynum venetum* L. and *Eleutherococcus senticosus* to ensure the quality of the formulas. A rapid approach for simultaneous determination of hyperoside, isoquercitrin and eleutheroside E in *Apocynum venetum* L. and *Eleutherococcus senticosus* by high-performance liquid chromatography with a diode array detector was described and validated. The full method validation, including the linearity, limits of detection and quantification, precision, repeatability, stability and recovery, was examined. All target components, including isomers of hyperoside and isoquercitrin, were baseline separated in 35 min. The developed method was sensitive, reliable and feasible. With this method, the optimal decoction conditions of *Apocynum venetum* L. and *Eleutherococcus senticosus* were selected, and their quality analysis was carried out. Furthermore, an herbal compatibility study of *Apocynum venetum* L. and *Eleutherococcus senticosus* based on detecting variations in the content of their active ingredients was performed by the developed HPLC method. It could be an alternative for the quantitative analysis of herbs that contain hyperoside, isoquercitrin or (and) eleutheroside E in the future.

## Introduction

Botanical herbs are consumed globally not only as an essential component of the diet but also as medicines or as functional food supplements. As herbs used in traditional Chinese medicine (TCM), *Apocynum venetum* L. (AV) and *Eleutherococcus senticosus* (ES), also named *Acanthopanax senticosus* (Rupr. et Maxim.) Harms, have been used for hundreds of years to treat hypertension in China. In addition to reducing blood pressure, the formulas can also be used to relieve stroke and other heart diseases [1]. Based on the multifold efficacy, an increasing number of patients with hypertension take these herbs. Thus, it is urgent and essential to develop modern analytical methods to ensure the quality of the formulas.

*Apocynum venetum* L. (Luobuma in Chinese) is a perennial herbaceous or half-shrub plant that grows in central to northwestern China [2]. *Apocynum venetum* L. has been used as an “antihypertensive tea” in China and Japan. It appears to be a popular beverage all over the world [3]. Previous studies reported AV has some pharmacological activities, such as antioxidants, [3–5] anti-hypertensive [1, 6] and anti-depressant effects, [7, 8] cholesterol lowering [1] and anti-diabetic activity [9]. It has been shown to lower blood pressure in vivo and cause in vitro vasodilatation of rat aortic and mesenteric arterial rings [6, 10]. AV is rich in minerals and flavonoids, and its main active fractions are found to be phenolic acid, flavone and flavan-3-ol components [11, 12]. Among them, hyperoside and isoquercitrin are the main effective components [13]. New reports have demonstrated that hyperoside and isoquercitrin exhibit potent antioxidant activities, anti-hypertensive and cardiovascular protection [14, 15]. Therefore, hyperoside and isoquercitrin must be measured in the formulas.

*Eleutherococcus senticosus* (Ciwujia in Chinese), a shrub native to the taiga of China, Korea, Russia and Hokkaido Island of Japan, has been used as an adaptogen [16]. Currently, there are some ES products, including drugs and health food, on the market in many countries [16, 17]. In vitro and in vivo studies have demonstrated that ES possesses many pharmacological effects, such as antistress, antifatigue and antidepressive effects [18]. It also has beneficial effects on hypertension, ischemic heart disease, chronic bronchitis, autoimmune diseases, gastric ulcers and allergic responses [19]. It has been reported that ES has some active constituents, including lignans (eleutheroside E), glycans (eleutheroside D), triterpene saponins (eleutherosides I, K, L and M), steroid glycosides (eleutheroside A), hydroxycoumarins, phenylacrylic acid derivatives and flavones [20, 21]. Eleutheroside E is known to reduce physical fatigue and to enhance endurance performance [21]. According to previous research results, eleutheroside E is the major component attributed to the pharmacological effects of ES [20, 21]. Therefore, eleutheroside E should be detected in the formulas.

**Fig. 1:**
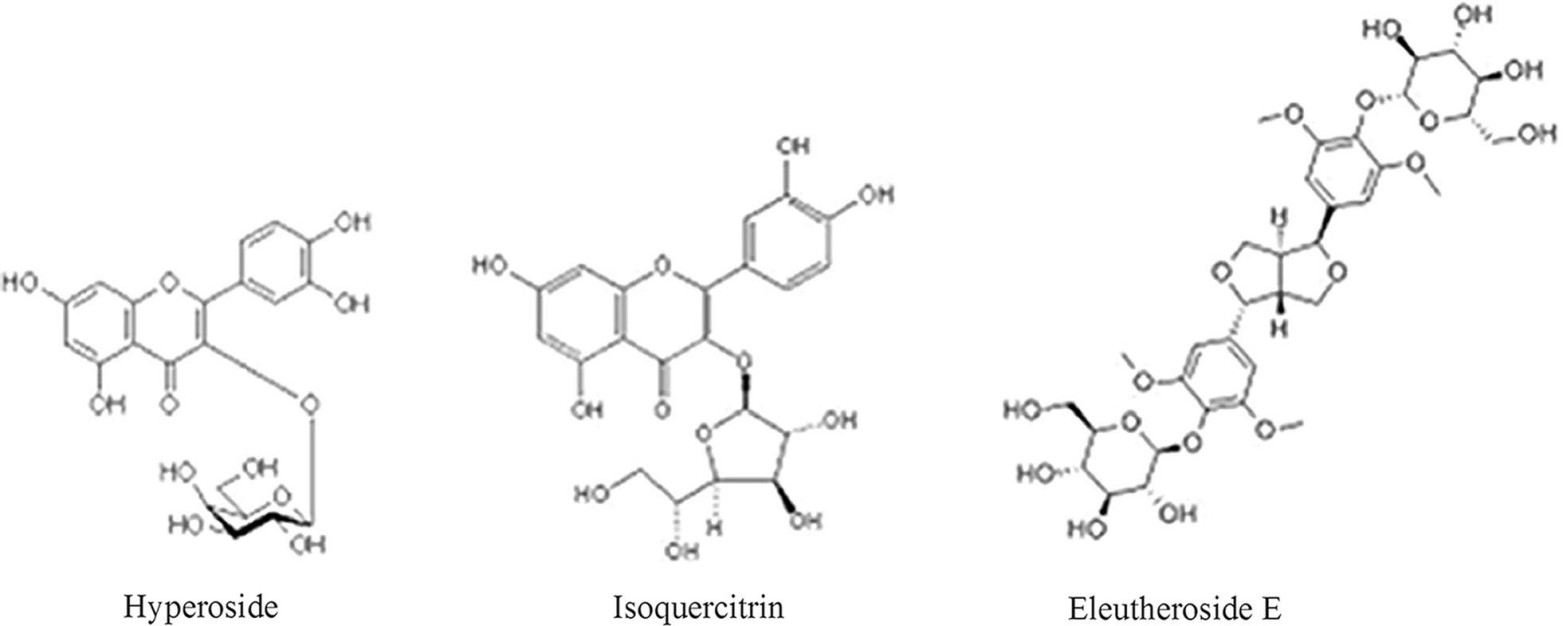
Chemical structures of hyperoside, isoquercitrin and eleutheroside E

In previous research, hyperoside and isoquercitrin in AV were simultaneously separated and detected by mixed cloud point extraction (MCPE) combined with HPLC [22]. A sensitive LC–MS-MS method for simultaneous quantification of hyperoside and isoquercitrin was also reported [23]. Some studies have also reported analytical methods for eleutheroside E detection by HPLC [24]. However, until now, no article has reported the simultaneous detection of hyperoside, isoquercitrin and eleutheroside E (Fig. 1). The quantification of hyperoside and isoquercitrin by HPLC–MS or MCPE-HPLC is complicated and not suitable for quality control. Therefore, this study aims to develop an efficient method for simultaneous separation and determination of hyperoside, isoquercitrin and eleutheroside E and apply it in quality control of formulas of AV and ES.

## Materials and methods

### Chemicals and reagents

Standard substances for content determination, including hyperoside (Batch No. 111521-201406), isoquercitrin (Batch No. 111809-201403) and eleutheroside E (Batch No. 111713-200502), were purchased from the National Institutes for Food and Drug Control (Beijing, China). The Chinese herbs *Apocynum venetum* L. and *Eleutherococcus senticosus* were obtained from Huadong Medicine Co., Ltd. (Hangzhou, China). Chinese medicine granules of *Apocynum venetum* L. (Batch No. 1512617) and *Eleutherococcus senticosus* (Batch No. 1507077) were kindly supplied by Guangdong Yifang Pharmaceutical Co., Ltd. China (Foshan, China) and Jiangyin Tianjiang Pharmaceutical Co., Ltd. (Jiangyin, China), respectively. HPLC-grade acetonitrile and phosphoric acid were purchased from Merck (Darmstadt, Germany) and Sigma (Fairfield, USA), respectively. The deionized water used throughout the experiments was produced using a Milli-Q water purification system (Milford, MA, USA).

### Sample preparation

#### Standard solution

The standard stock solution of hyperoside (2.0 mg/mL), isoquercitrin (2.0 mg/mL) and eleutheroside E (2.0 mg/mL) was prepared in methanol and stored at 4 °C. Working solutions with the lower concentrations were prepared by an appropriate dilution of the stock solution.

#### Decoctions of AV and ES

Herbs AV and ES were purchased from the Chinese Medicine Factory of Zhejiang University of Traditional Chinese Medicine (Hangzhou, China). The herbs were obtained in September and identified by professor Zheng. The leaves of AV and the roots of ES were taken for preparation of the decoction. Five milligrams of AV and 10 mg ES were soaked in water together and decocted in marmite. The decocting conditions (L_9_ 3 4 orthogonal assay) are listed in Table 1. The decoctions were filtered and metered to 250 mL with water. The decoctions were concentrated to a powder by lyophilization. It was stored at 4 °C until analysis.

**Table 1:** Decoctions of *Apocynum venetum* L. and *Eleutherococcus senticosus* prepared under different decocting conditions

Decoctions	Soaking time (h)	Water addition (volumes)	Decoction duration (min)	Frequency of decoction (frequency)
1	0.5	10	10	1
2	0.5	12	15	2
3	0.5	15	20	3
4	1	10	15	3
5	1	12	20	1
6	1	15	10	2
7	2	10	20	2
8	2	12	10	3
9	2	15	15	1

#### Preparation process of electuary of AV and ES formulas

Chinese herbs of 2 kg AV and 4 kg ES were mixed. Fifteen volumes of water were added to the mixture. The mixture was soaked for 0.5 h. It was decocted 3 times for 20 min per time. The decoctions were mixed, filtered and concentrated to a relative density of 1.2. Then, the decoction was processed to an electuary by marumerization. The dosage of cyclodextrin was 400 g in the marumerization.

#### Samples prepared for HPLC analysis

Ten milligrams of powder decoctions of AV and ES, 10 mg electuary of AV and ES, and 10 mg granules of AV and (or) ES were all diluted with 1 mL methanol. These samples were prepared by centrifugation (12000 rpm for 15 min) at 4 °C. Then, the supernatants were acquired for HPLC analysis.

### Instrumentation and analytical conditions

HPLC analyses were performed on an Agilent 1260 series system (Agilent Technologies, USA) consisting of a quaternary pump, online vacuum degasser, autosampler, thermostated column compartment and DAD full-wavelength scanning detector. An Agilent TC-C18 column (150 mm × 4.6 mm i.d., 5.0 μm particle size) from Agilent Technologies (USA) was used for all chromatographic separations. A linear gradient elution of Eluents A (0.1% (v/v) aqueous phosphoric acid) and B (0.1% (v/v) phosphoric acid in acetonitrile) was used for the separation. The elution program was well optimized and conducted as follows: the first linear gradient was 5% Eluent B in the range of 0 ~ 3 min, the second one was 5% ~ 12% Eluent B in the range of 3 ~ 8 min, the third one was 12% ~ 15% Eluent B in the range of 8 ~ 11 min, the fourth one was 15% Eluent B in the range of 11 ~ 25 min and the last one was 15% ~ 100% Eluent B in the range of 25 ~ 35 min. Then, the system was restored to the initial conditions after 5 min. The solvent flow rate was 1.0 mL/min, the detection wavelength was set at 210 nm, the column temperature was maintained at 25 °C and the injection volume was 20 μL. Chemstation software (Agilent Technology) was used for peak detection and peak area calculation.

### Method validation

#### Linearity and range

The linearity and range of the analytical assay were determined by serial dilution of a standard stock solution. Standard calibration curves were generated by calculating the ratios between the chromatographic peak area of each standard substance and the corresponding concentration.

#### Limit of quantification (LOQ) and limits of detection (LOD)

The LOQ and LOD were both determined using a signal-to-noise approach. LOQ was defined as the lowest concentration level resulting in a peak height of 10 times the baseline noise (the signal-to-noise ratio (S/N) is 10). LOD was defined as the minimum concentration that could be calculated at S/N = 3.

#### Precision, repeatability and stability

The precision was determined by analyzing the same concentration solutions of standards five consecutive times with the established HPLC method. The repeatability was determined using five duplicate samples from the powder of the same batch decoction, treated according to the sample preparation procedure (2.2.4) and analyzed with the established HPLC method. The stability was determined by testing the same powder decoction sample at six time points (0, 3, 6, 9, 12 and 24 h) over 24 h with the established HPLC method.

#### Recovery test

In the recovery test, three different quantities (low, medium and high) of standards were added to the same powder decoction sample. Then, the mixture was treated according to the sample preparation procedure (2.2.4) and analyzed by the developed HPLC method. Then, the quantity of each component was subsequently calculated from the corresponding calibration curve.

## Results and discussion

### Chromatographic separation

**Fig. 2:**
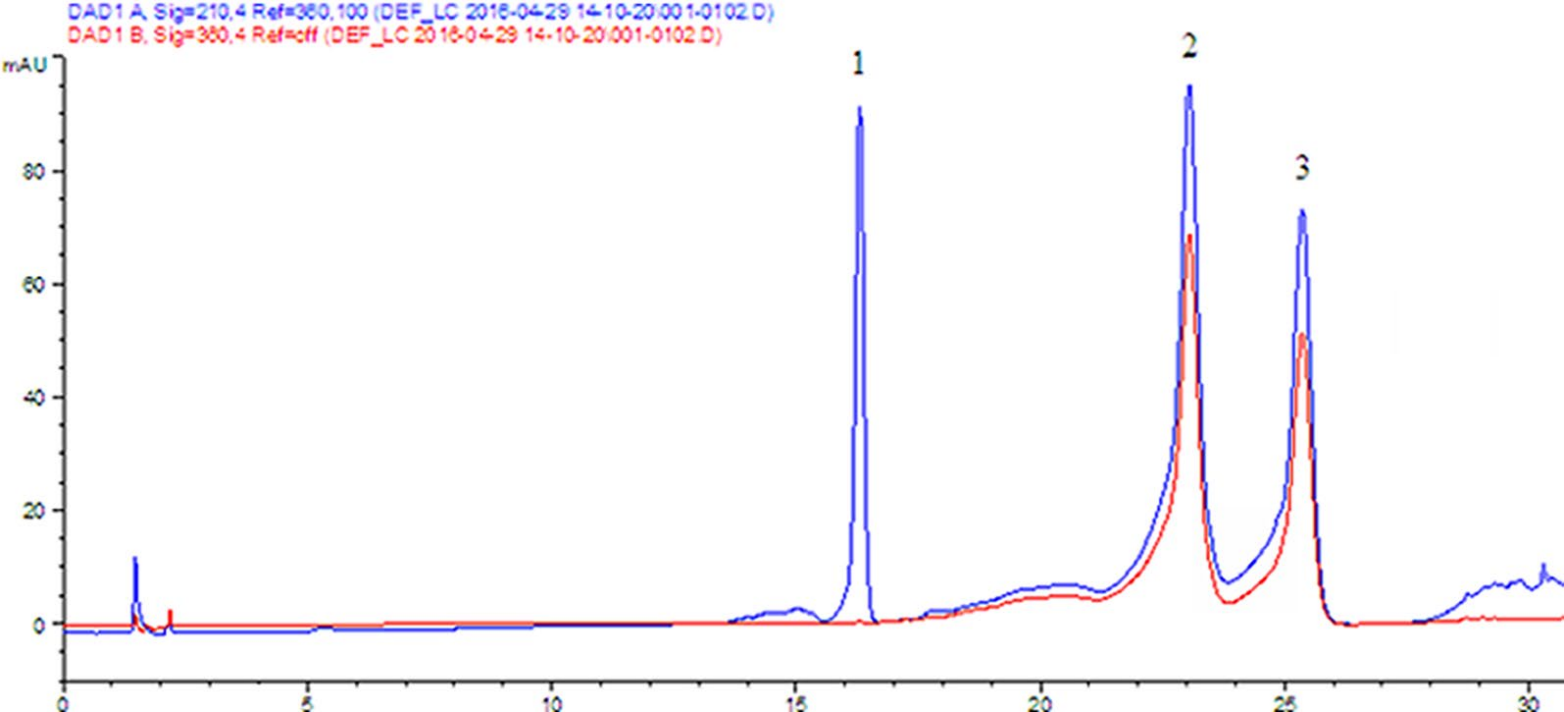
HPLC chromatograms of 0.05 mg/mL standard solutions of hyperoside, isoquercitrin and eleutheroside E at 210 nm (blue chromatogram) and 360 nm (red chromatogram) separately. 1. Eleutheroside E, 2. Hyperoside, 3. Isoquercitrin

**Fig. 3:**
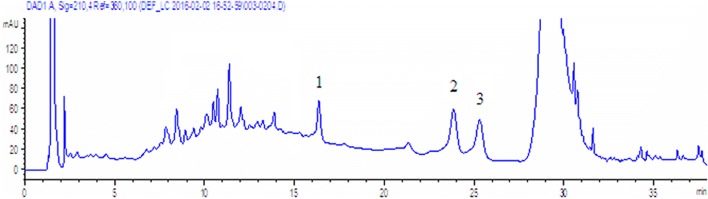
HPLC chromatogram of the electuary of *Apocynum venetum* L. and *Eleutherococcus senticosus* formulas under the optimized chromatographic conditions. 1. Eleutheroside E, 2. Hyperoside, 3. Isoquercitrin

**Fig. 4:**
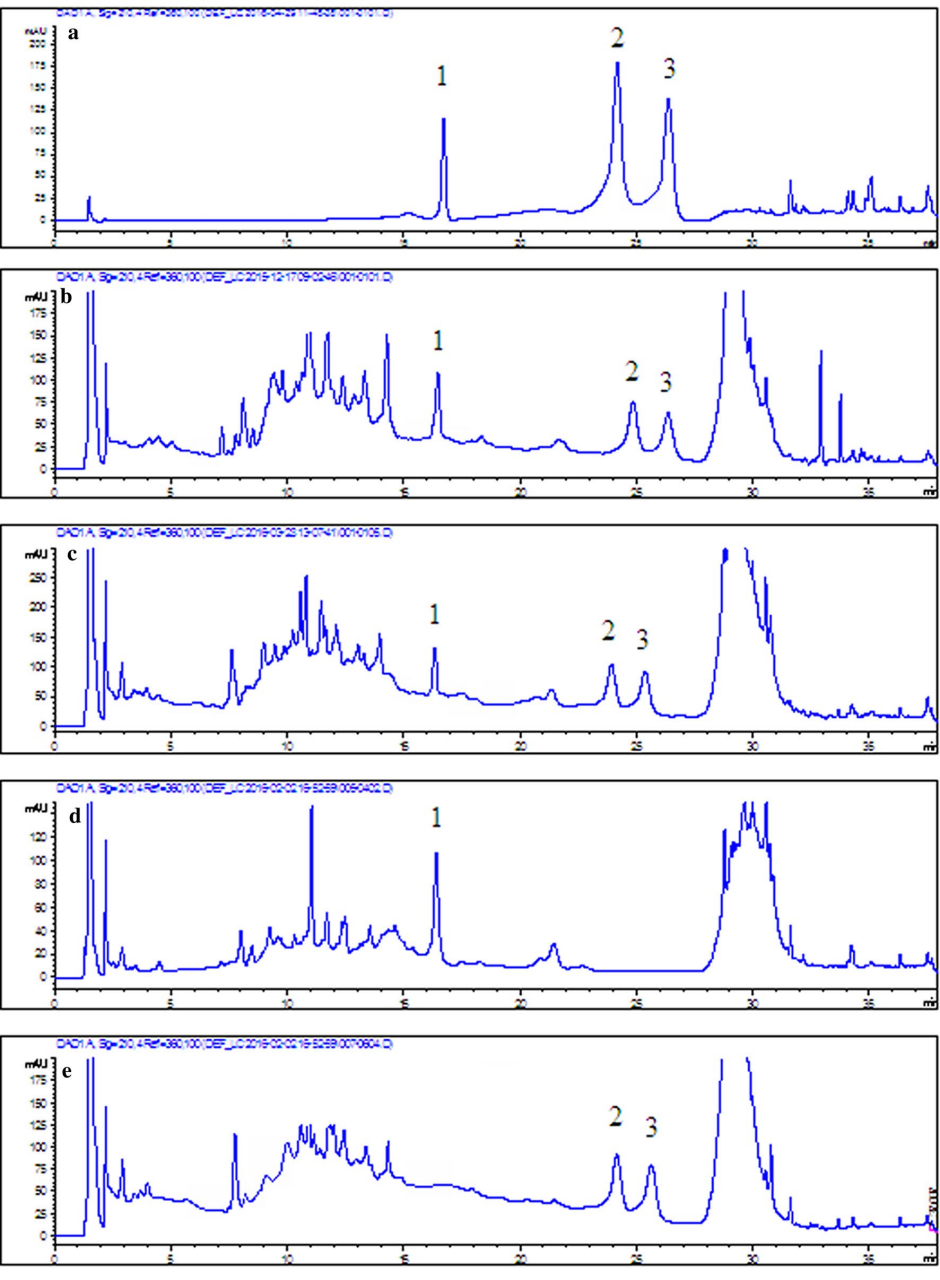
HPLC chromatograms of the standard solution and sample solution under the optimized chromatographic conditions. A. mixed standard solution, B. decoction of *Apocynum venetum* L. and *Eleutherococcus senticosus*, C. granules of *Apocynum venetum* L. and *Eleutherococcus senticosus*, D. granules of *Eleutherococcus senticosus*, E. granules of *Apocynum venetum* L. 1. eleutheroside E, 2. hyperoside, 3. isoquercitrin

An aqueous acetonitrile solvent system was used to analyze of hyperoside and isoquercitrin by HPLC. Water (0.1% (v/v) phosphoric acid) and acetonitrile were selected as the mobile phases, and 360 nm was selected as the detection wavelength in Shi’s experiment [25]. Another study reported a microemulsion mobile phase consisting of 2.5% (v/v) n-butanol, 1.2% (v/v) Genapol X-080, 0.5% (v/v) ethyl acetate and 95.8% (w/v) aqueous 20 mM phosphoric acid, and 360 nm was selected to detect six flavonoids of *Apocynum venetum* L. extract, including hyperoside and isoquercitrin [26]. Fan [27] used water (0.5% (v/v) trifluoroacetic acid) and acetonitrile as the mobile phases and, 210 nm as the detection wavelength to detect eleutheroside E. According to the above literature, water (0.1% (v/v) phosphoric acid) and acetonitrile (0.1% (v/v) phosphoric acid) were selected as the mobile phases in our assay, and 210 nm and 360 nm were both investigated to determine which one was better for detecting hyperoside, isoquercitrin and eleutheroside E simultaneously. As seen in the chromatograms (Fig. 2), hyperoside and isoquercitrin had larger responses at 210 nm than at 360 nm. Furthermore, 210 nm was suitable wavelength for the determination of eleutheroside E, reported in the literature, as no response was observed at 360 nm for eleutheroside E. Therefore, 210 nm was selected as the detection wavelength for simultaneous determination of hyperoside, isoquercitrin and eleutheroside E. The column temperature was set at 25 °C, and the flow rate was 1.0 mL/min for the recommended chromatographic column. To rapidly separate hyperoside, isoquercitrin and eleutheroside E, a gradient elution procedure was carried out. Eleutheroside E was separated first without interference from hyperoside or isoquercitrin. However, the chromatographic peaks of hyperoside and isoquercitrin were not cleanly separated because of isomerization. Thus, isocratic elution with 15% eluent B was performed until the baseline separation of hyperoside and isoquercitrin was achieved. As shown in Fig. 3, when the running time was 23–25 min, hyperoside and isoquercitrin were separated. The gradient elution was then repeated. As seen from the above results, adjusting the mobile phase gradient and the time allowed the target compounds to achieve baseline separation without interference with each other. Finally, chromatograms of the standard solution and sample solution under the optimized chromatographic conditions are described in Fig. 4. Hyperoside, isoquercitrin and eleutheroside E were separated and detected rapidly and simultaneously by the developed method.

### Calibration curves, LOQ and LOD

Six concentrations (0.5, 1.0, 5.0, 10.0, 50.0, 100.0 μg/mL) of standard eleutheroside E solutions and seven concentrations (0.5, 1.0, 5.0, 10.0, 50.0, 100.0, 200 μg/mL) of mixed standard hyperoside and isoquercitrin solutions were prepared in quintuplicate to generate calibration curves. The integrated chromatographic peak areas (*Y*) were plotted against the corresponding concentrations (*X*, μg/mL) of the three constituents in the standard solutions to obtain calibration curves based on linear regression analysis. Satisfactory linearity was obtained, as shown by the correlation coefficients higher than 0.999 in the investigated ranges (Table 2). The LOD (*S/N *= 3) value for three target components was 0.1 μg/mL. The LOQ (*S/N *= 10) value for three target components was 0.5 μg/mL, which met the sensitivity requirement for quantitative analysis.

**Table 2:** Results of regression analysis on calibration curves and limits of detection (*n *= 5)

Compound	Test range (μg/mL)	Calibration curves	*R*	LOQ (μg/mL)	LOD (μg/mL)
Hyperoside	0.50–200.00	Y = 52.995X − 86.148	0.9996	0.50	0.10
Isoquercitrin	0.50–200.00	Y = 40.714X − 90.563	0.9994	0.51	0.12
Eleutheroside E	0.50–100.00	Y = 24.193X +7.4352	0.9998	0.50	0.10

### Precision, repeatability and stability

Various concentrations (1.0, 10.0 and 100.0 μg/mL) of mixed standard solutions of hyperoside, isoquercitrin and eleutheroside E were injected and analyzed in quintuplicate by HPLC to determine the precision of the method. The precision was evaluated by the RSD values of the peak areas of the three components, which ranged from 0.61 to 1.19% (hyperoside), 0.37% to 0.87% (isoquercitrin) and 0.69% to 1.81% (eleutheroside E). The results confirmed that the HPLC method had good precision.

The repeatability was evaluated by the RSD values of the contents of hyperoside, isoquercitrin and eleutheroside E in five duplicate samples from powders of the same batch decoction. The RSD values were 0.42% for hyperoside, 1.20% for isoquercitrin and 2.42% for eleutheroside E, which demonstrated the good repeatability of this method.

The stability test of the powder decoction sample gave a good result. The RSD values of contents of the hyperoside, isoquercitrin and eleutheroside E were 0.73%, 0.97% and 0.77%, respectively, showing that the method had good stability for the determination of hyperoside, isoquercitrin and eleutheroside E in 24 h.

### Recovery test

A sample of decoction powder was detected by the proposed HPLC method. The contents of hyperoside, isoquercitrin and eleutheroside E in this sample were 30.63, 37.23 and 42.32 μg/mL, respectively. Then, three different quantities (low, medium and high) of standards were added to this sample. The recovery was evaluated by the following formula: Recovery = (amount found- amount sample)/amount standard spiked × 100%. The results are shown in Table 3. The recoveries ranged from 97.82 to 104.54%, which validated the accuracy of the method.

**Table 3:** Recovery test of the developed HPLC analysis method (*n *= 5)

Compound	Standard spiked (μg/mL)	Found (μg/mL)	Recovery (%)	Average recovery (%)	RSD (%)
Hyperoside	24.50	60.72 ± 0.32	122.82	104.54	16.13
	30.63	61.62 ± 0.26	101.18		
	36.75	63.56 ± 0.56	89.61		
Isoquercitrin	29.78	70.60 ± 0.86	112.06	104.53	8.06
	37.23	76.73 ± 0.51	106.10		
	44.68	79.87 ± 0.56	95.43		
Eleutheroside E	33.86	77.17 ± 0.68	102.91	97.82	6.21
	42.32	84.41 ± 0.48	99.46		
	50.78	88.58 ± 0.49	91.10		

### Sample analysis

#### Analysis of decoction engineering of the formulas of *Apocynum venetum* L. and *Eleutherococcus senticosus*

The addition of water, soaking time, decoction duration and frequency of decoction were the major factors in the herbal decoction procedure. These four factors and three-level orthogonal experiments of decoction engineering were analyzed by determining the contents of hyperoside, isoquercitrin and eleutheroside E. The data acquired were analyzed by ANOVA to obtain the optimal decoction conditions of the formulas of AV and ES. First, the content of hyperoside was selected as the dependent variable, and the mean square values of the four factors were 9.456 (soaking time), 4.366 (water addition), 9.121 (decoction duration) and 5.502 (frequency of decoction). Water addition was the factor with the least effect. Thus, it was regarded as the blank control to assess the other factors. The results showed that the *P* values were 0.316, 0.324 and 0.442 for the factors of soaking time, decoction duration and frequency of decoction, respectively. There were no significant differences among these factors when the hyperoside content was considered the dependent variable. Similarly, the content of isoquercitrin was selected as the dependent variable, and the mean square values of the four factors were 3.708 (soaking time), 1.969 (water addition), 9.693 (decoction duration) and 2.450 (frequency of decoction), respectively. Water addition was selected as the blank control because it had the lowest mean square value among the four factors. Then, the *P* values were obtained by ANOVA, 0.347 for soaking time, 0.169 for decoction duration and 0.446 for frequency of decoction. There were no significant differences among these factors when the content of isoquercitrin was considered the dependent variable. Finally, the content of eleutheroside E was selected as the dependent variable in the assay. The results are shown in Table 4. Soaking time was selected as the blank control because it had the lowest mean square value among the four factors. The frequency of decoction had a greater impact on the content of eleutheroside E than the other factors. Moreover, the frequency of decoction obviously affected the content of eleutheroside E, with a *P* value of 0.030 (*P *< 0.05, significant difference). It was illustrated that the frequency of decoction had a significant influence on the decoction engineering of the formulas of AV and ES. Therefore, three frequencies of decoction were recommended in decoction engineering. Considering the production efficiency, a soaking time of 0.5 h was determined to be suitable. It was suggested that a soaking time of 0.5 h, water addition of 15 volumes, a decoction duration of 20 min and a frequency of decoction of 3 would be the optimal decoction conditions, as evident from decoction engineering analysis based on the four factors and three-level orthogonal experiment and ANOVA results.

**Table 4:** The four factors and three-level orthogonal experiment and ANOVA results for the determination of eleutheroside E in decoctions of the formulas of AV and ES

	Sum of squares	df	Mean square	F	*P* (Sig.)
Calibration model	96.34	6	16.06	21.73	0.05
Intercept	11733.95	1	11733.95	15880.77	0
Water addition	22.35	2	11.18	15.13	0.06
Decoction duration	26.50	2	13.25	17.93	0.05
Frequency of decoction	47.49	2	23.75	32.14	0.03
Error (Soaking time)	1.48	2	0.74		

#### Determination of the electuary of the formulas of *Apocynum venetum* L. and *Eleutherococcus senticosus*

The optimal decoction conditions of formulas of AV and ES were selected. Thus, the electuary of the formulas of AV and ES was prepared under optimal decoction conditions. Determination of hyperoside, isoquercitrin and eleutheroside E was performed by the developed HPLC method to perform quality control of the electuary of the formulas of AV and ES. The results are shown in Table 5. The RSD values of hyperoside, isoquercitrin and eleutheroside E from different batches were 3.285%, 3.695% and 3.749%, respectively. The contents differed slightly among batches. It was demonstrated that the preparation of the electuary of the formulas of AV and ES was stable and feasible. Furthermore, the developed HPLC method was suitable for quality control of the electuary of the formulas of AV and ES.

**Table 5:** Determination of hyperoside, isoquercitrin and eleutheroside E in the electuary of the formulas of AV and ES by the developed HPLC method (*n *= 3)

Batch no.	Content of hyperoside (μg/mL)	Content of isoquercitrin (μg/mL)	Content of eleutheroside E (μg/mL)
1,512,201	25.24 ± 0.55	26.98 ± 0.47	20.81 ± 0.23
1,512,232	26.94 ± 0.43	28.89 ± 0.36	22.15 ± 0.15
1,512,291	25.26 ± 0.57	26.82 ± 0.46	20.69 ± 0.27
1,512,293	26.80 ± 0.48	28.77 ± 0.39	22.23 ± 0.19
1,512,302	26.68 ± 0.42	28.68 ± 0.35	22.30 ± 0.18

#### Study on the combination of *Apocynum venetum* L. and *Eleutherococcus senticosus*

**Fig. 5:**
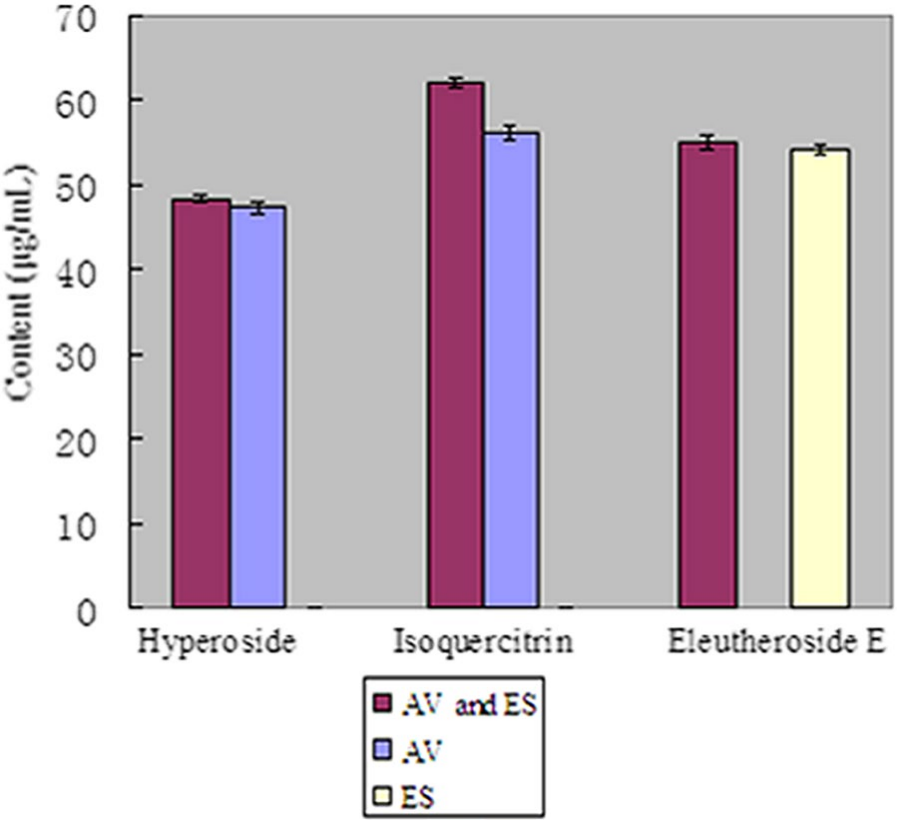
The contents of hyperoside, isoquercitrin and eleutheroside E in AV and ES granules, AV granules, and ES granules

The Chinese herbs AV and ES have been prescribed together to treat hypertension and cardiovascular disease. To prove the advantage of the combination of AV and ES, the effective components of AV and ES should be detected and analyzed by the developed HPLC method. Granules of AV and ES both combined and separate were studied in the assay. The results are shown in Fig. 5. The contents of hyperoside, isoquercitrin and eleutheroside E increased when AV and ES granules were dissolved together; in particular, the isoquercitrin content increased by 10.63%. The combination of AV and ES was beneficial for increasing the content of effective components in AV and ES, and thus, it improved the efficacy. The developed HPLC method was useful and rapid in the study of the combination of AV and ES.

## Conclusion

In this paper, an optimized HPLC analytical method was established and validated for the simultaneous determination of hyperoside, isoquercitrin and eleutheroside E in AV and ES. All target components including isomers of hyperoside and isoquercitrin were baseline separated within 35 min by simple HPLC–DAD rather than HPLC–MS. The developed method was applied to optimize the decoction engineering of AV and ES, perform quality control of the electuary of AV and ES and research the herbal combination of AV and ES. The method was sensitive, rapid and reliable; thus, it could be an alternative for future quantitative analysis of herbs that contain hyperoside, isoquercitrin or (and) eleutheroside E.

## Data Availability

We declared that materials described in the manuscript, including all relevant raw data, will be freely available to any scientist wishing to use them for non-commercial purposes, without breaching participant confidentiality.

## Abbreviations

AV: *Apocynum venetum* L
ES: *Eleutherococcus senticosus*
HPLC-DAD: High-performance liquid chromatographic with diode array detector
LOD: Limit of detection
LOQ: Limit of quantification
TCM: Traditional Chinese medicine
MCPE: Mixed cloud point extraction
S/N: Signal-to-noise ratio

## References

[1] Xie W, Zhang X, Wang T, Hu J (2012). Botany, traditional uses, phytochemistry and pharmacology of *Apocynum venetum L*. (Luobuma): a review. J Ethnopharmacol.

[2] Liang T, Yue W, Li Q (2010). Comparison of the phenolic content and antioxidant activities of *Apocynum venetum L*. (Luo-Bu-Ma) and two of its alternative species. Int J Mol Sci.

[3] Chan CO, Lau CC, Ng YF, Xu LJ, Chen SB, Chan SW, Mok DK (2015). Discrimination between leave of *Apocynum venetum* and its adulterant, *A. pictum* based on antioxidant assay and chemical profiles combined with multivariate statistical analysis. Antioxidants.

[4] Cao Y, Chu Q, Ye J (2003). Determination of hydroxyl radical by capillary electrophoresis and studies on hydroxyl radical scavenging activities of Chinese herbs. Anal Bioanal Chem.

[5] Shirai M, Kawai Y, Yamanishi R, Terao J (2005). Approach to novel functional foods for stress control 5. antioxidant activity profiles of antidepressant herbs and their active components. J Med Invest.

[6] Kim D, Yokozawa T, Hattori M, Kadota S, Namba T (2000). Effects of aqueous extracts of *Apocynum venetum* leaves on spontaneously hypertensive, renal hypertensive and NaCl-fed-hypertensive rats. J Ethnopharmacol.

[7] Butterweck V, Nishibe S, Sasaki T, Uchida M (2001). Antidepressant effects of apocynum venetum leaves in a forced swimming test. Biol Pharm Bull.

[8] Zheng M, Liu C, Pan F, Shi D, Zhang Y (2012). Antidepressant-like effect of hyperoside isolated from *Apocynum venetum* leaves: possible cellular mechanisms. Phytomedicine.

[9] Yokozawa T, Nakagawa T (2004). Inhibitory effects of Luobuma tea and its components against glucose-mediated protein damage. Food Chem Toxicol.

[10] Kwan C, Zhang W, Nishibe S, Seo S (2005). A novel in vitro endothelium-dependent vascular relaxant effect of *Apocynum venetum* leaf extract. Clin Exp Pharmacol Physiol.

[11] Han LW, Hou JJ, Zhao L, Liang TG, Li QS (2008). Establishment of HPLC fingerprint and its application in identification of Folium Apocyni Veneti. Chin Tradit Herb Drugs.

[12] An HJ, Wang H, Lan YX, Hashi Y, Chen SZ (2013). Simultaneous qualitative and quantitative analysis of phenolic acids and flavonoids for the quality control of *Apocynum venetum L*. leaves by HPLC-DAD-ESI-IT-TOF-MS and HPLC-DAD. J Pharm Biomed Anal.

[13] Zhou C, Sun L, Bi K (2009). RP-HPLC analysis of hyperoside and isoquercitrin in *Apocynum venetum L*. Chin J Pharm Anal.

[14] Piao X, Mi X, Tian Y, Wu Q, Piao H, Zeng Z, Wang D, Piao X (2009). Rapid identification and characterization of antioxidants from *Ligularia fischeri*. Arch Pharm Res.

[15] Shibano M, Kakutani K, Taniguchi M, Yasuda M, Baba K (2008). Antioxidant constituents in the dayflower (*Commelina communis L*.) and their alpha-glucosidase-inhibitory activity. J Nat Med.

[16] Davydov M, Krikorian AD (2000). Eleutherococcus senticosus (Rupr. and Maxim.) Maxim. (Araliaceae) as an adaptogen: a closer look. J Ethnopharmacol.

[17] Weng S, Tang J, Wang G, Wang X, Wang H (2007). Comparison of the addition of siberian ginseng (*Acanthopanax senticosu*s) versus fluoxetine to lithium for the treatment of bipolar disorder in adolescents: a randomized, double-blind trial. Curr Ther Res Clin Exp.

[18] Deyama T, Nishibe S, Nakazawa Y (2001). Constituents and pharmacological effects of Eucommia and Siberian ginseng. Acta Pharmacol Sin.

[19] Takahashi Y, Tanaka M, Murai R, Kuribayashi K, Kobayashi D, Yanagihara N, Watanabe N (2014). Prophylactic and therapeutic effects of *Acanthopanax senticosus* harms extract on murine collagen-induced arthritis. Phytother Res.

[20] Jung CH, Ahn J, Heo SH, Ha TY (2014). Eleutheroside E, an active compound from *Eleutherococcus senticosus*, regulates adipogenesis in 3T3-L1 cells. Food Sci Biotechnol.

[21] Ahn J, Um MY, Lee H, Jung CH, Heo SH, Ha TY (2013). Eleutheroside E, an active component of *Eleutherococcus senticosus*, ameliorates insulin resistance in type 2 diabetic db/db Mice. Evid Based Complement Alternat Med.

[22] Zhou J, Sun JB, Xu XY, Cheng ZH, Zeng P, Wang FQ, Zhang Q (2015). Application of mixed cloud point extraction for the analysis of six flavonoids in *Apocynum venetum* leaf samples by high performance liquid chromatography. Pharm Biomed Anal.

[23] Zhou C, Liu Y, Su D, Gao G, Zhou X, Sun L, Ba X, Chen X, Bi K (2011). A sensitive LC–MS–MS method for simultaneous quantification of two structural isomers, hyperoside and isoquercitrin: application to pharmacokinetic studies. Chromatographia.

[24] Yang L, Ge H, Wang W, Zu Y, Yang F, Zhao C, Zhang L, Zhang Y (2013). Development of sample preparation method for eleutheroside B and E analysis in *Acanthopanax senticosus* by ionic liquids-ultrasound based extraction and high-performance liquid chromatography detection. Food Chem.

[25] Shi Q, Deng F, Wu M (2014). Simultaneous determination of rutin, hyperin and isoquercitrin in leaves of *Apocynum venetum* and *Poacynum hendersonii* located in Xinjiang by HPLC. Chin Tradit Herb Drugs.

[26] Song R, Zhou J (2015). Microemulsion liquid chromatographic method for simultaneous separation and determination of six flavonoids of *Apocynum venetum* leaf extract. J Chromatogr B.

[27] Fan R, Fu H, Jin X, Wang J, Gao C, Gai C, Hu R (2014). Extraction technology for active constituents in Acanthopanax senticosus and comparison on its contents of *A. senticosus* from various habitats. Chin Tradit Herb Drugs.

